# Report of a Case of Inflammation of the Fauces and Glottis, Resulting in Complete Obstruction of Respiration; Tracheotomy Successfully Performed

**Published:** 1849-02

**Authors:** 


*Report of a Case of Inflammation of the Fauces and Glottis, resulting in Complete Obstruction of Respiration; Tracheotomy successfully performed.* By J. B. HERRICK, M. D., Demonstrator of Anatomy in Rush Medical College.

I was called, at 2 o'clock, A. M., of Aug. 8, 1847, to visit Frederick P., æt. four years. The father of the patient—who called me—seemed much alarmed; and, upon entering his house, I found his fears well grounded. The respiration of the child, which was so difficult and labored as not to permit him to assume any but the erect position, could be heard distinctly in distant apartments of the dwelling, and seemed to threaten immediate suffocation.

From the parents, I learned the following history of the case:—About three weeks previously, they had noticed that the child manifested a sensation of pain when he swallowed portions of solid food; subsequently, a swelling appeared about the angles and base of the lower jaw, and still more recently, difficulty of breathing had been superadded, which latter symptom had existed ten days, and had been slowly but constantly increasing in severity, especially during each night. The excuse given by the parents for not calling aid sooner was, that "they had suspected the malady to be mumps, and thought their child would recover without medical aid." I found the patient in a high state of febrile excitement, with a swelling of the glands, similar in appearance externally to what would occur, in a well marked case of cynanche parotidæ. Upon examining the mouth, I found the parts from and including the anterior palatine pillars and uvula, and extending as far back as I could discern, highly inflamed and much swollen: the uvula and anterior pillars so much so as to make the opening to the fauces quite small. I at once supposed the difficulty to be an abscess, or the lodgement of some foreign body, at the root of the tongue, and accordingly introduced my finger to detect it. I could, however, find nothing, and so far as the exploration could be carried, the swelling seemed to be general and uniform; even the tonsils did not assume more than a proportionate amount of the enlargement, to lead one to suspect them of having been the primary seat of the affection.

The epiglottis and rima glottidis—although the examination added so much to the difficulty of breathing, as of necessity to be made hastily and imperfectly—were found involved in such a state of inflammation and enlargement, as, together with physical signs, to leave no further doubt of the cause of the present urgent symptoms.

I commenced my course of treatment by scarifying the engorged parts; took a liberal quantity of blood from the temporal artery; gave an emetic of tart. ant., which had a good effect, and applied fomentations over the seat of the inflammation. A slight amelioration resulted from these remedies. I returned home, after prescribing fomentations, and a solution of tart. ant., to be given in sufficient quantity to produce slight nausea.

Returning again, after a few hours, I found my patient so much worse, that I thought proper to inform the parents that tracheotomy offered the only possible chance for relief. I explained to them the nature of the operation, and the uncertainty of its success. The case had now become so evidently critical that they readily gave their consent, and even urged me to hasten its performance, when they saw their child convulsed and in the agonies of approaching death from suffocation. The operation was gone through with in the ordinary manner without any untoward occurrence, save the escape of a small quantity of venous blood into the trachea, which was expelled by a few efforts at coughing as soon as the canula was inserted. The relief was immediate and most gratifying. The convulsions ceased as soon as air was freely admitted to the lungs, and in a few minutes, the patient fell into a quiet slumber. He was suffered to remain for several hours without being disturbed by the use of any other remedies.

I then directed a powder every third hour, composed of two grains of calomel, and one of ipecac., until three were given; to be followed by a dose of castor oil, three hours after the last powder. During the forenoon of the next day, he had several copious dark bilious evacuations, and the febrile symptoms gradually subsided, so that on the day following, the little fellow was so much better that he began to realize his situation, and was so chagrined with his new breathing apparatus, and at the loss of his voice, that he became very petulant, and could not be controlled by his parents, but had, during the remaining time he was under treatment, the liberty of the house and yard nearly as he pleased.

The subsequent treatment consisted in the use of a low diet; counter irritation, by the use of ung. ant. over the affected parts, and the daily application of a solution of arg. nit.—8 grains to the ounce—to the inflamed surface,

The swelling externally gradually subsided, and, at the expiration of eight days, appeared considerably less about the uvula and pillars of the palate; but still I found by closing the orifice of the canula, that air could not pass the superior opening of the larynx, and the removal of it would have resulted in instant suffocation. I now substituted the tinct. of iodine for the solution of nitrate of silver as an application to the inflamed surface, and continued the counter irritation as before.

After using this for ten days, without any perceptible improvement in the capacity of the larynx for the performance of its functions, the use of all local applications was discontinued. I now excised a small portion of each tonsil, which was accomplished with some difficulty, owing to the fact that they appeared but slightly enlarged, not sufficiently to obstruct respiration in the least. By this means, however, a small quantity of blood was abstracted from near the seat of the disease, and was doubtless beneficial.

I then directed 3 grains of iodide of potassium every third hour till 12 grains were given. This prescription was repeated for six days in succession. The third day ufter I commenced its use, I was gratified to find, by the return of the voice, when the orifice of the canula was closed, that the case was improving; and, at the end of eight days—twenty-six days after the operation—I removed the tube, and closed the artificial opening to the trachea with a strip of adhesive plaster. The wound healed rapidly, leaving but a slight scar; and since that time, the child has enjoyed good health, and the larynx and trachea have performed their functions perfectly.—*North Western Medical and Surgical Journal.*

